# β-Lactamase and Macrolide Resistance Gene Carriage in *Escherichia coli* Isolates Among Children Discharged From Inpatient Care in Western Kenya: A Cross-sectional Study

**DOI:** 10.1093/ofid/ofae307

**Published:** 2024-06-03

**Authors:** Polycarp Mogeni, Olusegun O Soge, Kirkby D Tickell, Stephanie N Tornberg, Rushlenne Pascual, Erika Wakatake, Mame M Diakhate, Doreen Rwigi, Kevin Kariuki, Samuel Kariuki, Benson O Singa, Ferric C Fang, Judd L Walson, Patricia B Pavlinac

**Affiliations:** Center for Microbiology Research, Kenya Medical Research Institute, Nairobi, Kenya; Department of Global Health, University of Washington, Seattle, Washington, USA; Department of Global Health, University of Washington, Seattle, Washington, USA; Division of Allergy and Infectious Diseases, Department of Medicine, University of Washington, Seattle, Washington, USA; Department of Laboratory Medicine and Pathology, University of Washington, Seattle, Washington, USA; Department of Global Health, University of Washington, Seattle, Washington, USA; The Childhood Acute Illness and Nutrition Network, Nairobi, Kenya; Department of Global Health, University of Washington, Seattle, Washington, USA; Department of Epidemiology, University of Washington, Seattle, Washington, USA; Department of Global Health, University of Washington, Seattle, Washington, USA; Department of Global Health, University of Washington, Seattle, Washington, USA; Department of Global Health, University of Washington, Seattle, Washington, USA; Center for Microbiology Research, Kenya Medical Research Institute, Nairobi, Kenya; Center for Microbiology Research, Kenya Medical Research Institute, Nairobi, Kenya; Center for Microbiology Research, Kenya Medical Research Institute, Nairobi, Kenya; Center for Microbiology Research, Kenya Medical Research Institute, Nairobi, Kenya; Department of Global Health, University of Washington, Seattle, Washington, USA; Division of Allergy and Infectious Diseases, Department of Medicine, University of Washington, Seattle, Washington, USA; Department of Laboratory Medicine and Pathology, University of Washington, Seattle, Washington, USA; Department of Microbiology, University of Washington, Seattle, Washington, USA; Department of Global Health, University of Washington, Seattle, Washington, USA; Division of Allergy and Infectious Diseases, Department of Medicine, University of Washington, Seattle, Washington, USA; The Childhood Acute Illness and Nutrition Network, Nairobi, Kenya; Department of Pediatrics, University of Washington, Seattle, Washington, USA; Department of Global Health, University of Washington, Seattle, Washington, USA; Department of Epidemiology, University of Washington, Seattle, Washington, USA

**Keywords:** β-lactamase, CTX-M, hospital, *mph*(A), routine vaccination

## Abstract

**Background:**

Antimicrobial resistance (AMR) is a global threat to infectious disease control, particularly among recently hospitalized children. We sought to determine the prevalence and mitigating factors of resistance in enteric *Escherichia coli* among children discharged from health facilities in western Kenya.

**Methods:**

Between June 2016 and November 2019, children aged 1 to 59 months were enrolled at the point of discharge from the hospital. *E coli* was isolated by microbiological culture from rectal swabs at baseline. β-Lactamases and macrolide resistance–conferring genes were detected by polymerase chain reaction. A modified Poisson regression model was used to assess the predictors *mph*(A) and CTX-M–type extended-spectrum β-lactamase (ESBL).

**Results:**

Of the 238 children whose *E coli* isolates were tested, 91 (38.2%) and 109 (45.8%) had detectable CTX-M–type ESBL and *mph*(A) genes, respectively. Antibiotic treatment during hospitalization (adjusted prevalence ratio [aPR], 2.47; 95% CI, 1.12–5.43; *P* = .025), length of hospitalization (aPR, 1.42; 95% CI, 1.00–2.01; *P* = .052), and the practice of open defecation (aPR, 2.47; 95% CI, 1.40–4.36; *P* = .002) were independent predictors for CTX-M–type ESBL and *mph*(A) genes. Pneumococcal vaccination was associated with a 43% lower likelihood of CTX-M–type ESBL (aPR, 0.57; 95% CI, .38–.85; *P* = .005), while measles vaccination was associated with a 32% lower likelihood of *mph*(A) genes (aPR, 0.68; 95% CI, .49–.93; *P* = .017) in *E coli* isolates.

**Conclusions:**

Among children discharged from the hospital, history of vaccination, shorter hospital stay, lack of in-hospital antibiotic exposure, and improved sanitation were associated with a lower likelihood of AMR genes. To mitigate the continued spread of AMR, AMR control programs should consider strategies beyond antimicrobial stewardship, including improvements in sanitation, increased vaccine coverage, and the development of novel vaccines.

Approximately 5 million deaths worldwide were associated with antimicrobial resistance (AMR) in 2019, 22% of which occurred in sub-Saharan Africa (SSA) [1]. Bacteria can acquire AMR through chromosomal mutations or horizontal exchange of genetic material [2]. The development and persistence of AMR are strongly associated with the inappropriate use of antimicrobial drugs [3, 4]. Infections caused by bacteria with AMR have been associated with prior inpatient admission, length of hospital stay, antimicrobial drug use, and hygiene [5–7]. Hospitalized children who acquire antibiotic-resistant infections present considerable treatment challenges [8], and this may partly explain the high postdischarge mortality reported in SSA, which ranges from 3% to 5% and is similar to inpatient mortality rates [1, 9, 10]. AMR acquired during hospitalization may predispose patients to treatment failure during the postdischarge period, thus elevating the likelihood of adverse outcomes [11]. In addition, prolonged antibiotic use due to AMR may alter the gut microbiota, which is associated with immune function, or it may increase the likelihood of resistance gene sharing from commensal bacteria to pathogenic bacteria [12].

*Escherichia coli* is a gram-negative bacterium that is a normal component of the human gastrointestinal microbiota with disease-causing potential [13]. *E coli*, including antibiotic-resistant *E coli*, can be spread from person to person in crowded environments or in settings with poor access to clean water and sanitation [14]. As a permanent resident of the gastrointestinal tract, AMR *E coli* may act as a reservoir for AMR genetic elements that can be transferred to other bacteria that reside in or pass through the intestinal tract [15]. Understanding the risk factors for exposure/acquisition of resistant bacteria can inform interventions to interrupt transmission. Vaccination, for example, has been shown to decrease not only the burden of infectious diseases but also AMR in controlled study settings [16–19]. However, the impact of routine vaccination on AMR bacteria in field studies is poorly documented, especially among children in SSA.

We previously described the prevalence of phenotypically determined resistance to commonly used antibiotics in *E coli* isolates and assessed the correlates of phenotypically determined extended-spectrum β-lactamase (ESBL)–producing *E coli* and Klebsiella isolates in a sample of children discharged from the hospital in western Kenya [5, 7]. In our previously reported cross-sectional survey, phenotypic AMR was high and associated with prior hospitalization, antibiotic use, sanitation, and hygiene variables [5]. However, phenotypic resistance testing does not provide information about potential mechanisms of AMR. Understanding the dynamics of resistant strains and resistance determinants is vital for the establishment of appropriate control measures. Here, we report the prevalence of genetic markers of macrolide and β-lactam resistance—2 important classes of antibiotics for common bacterial infections—among children living in SSA. We also report an exploratory analysis of the potentially modifiable correlates (including vaccination history) of CTX-M–type ESBL and *mph*(A): the most common genetic determinants of ESBL [20] and macrolide [21] resistance, respectively, in SSA. In addition, CTX-M–type ESBLs are the most prevalent and globally disseminated ESBLs in Enterobacteriaceae and confer cross-resistance to noncephalosporin classes of antimicrobials [22, 23].

## METHODS

### Patient Consent Statement

Ethical approval was provided by the institutional review boards of the Kenya Medical Research Institute (SERU 3086), the Kenyan Pharmacy and Poisons Board (PPB/ECCT/15/10/04), and the University of Washington (49120). The parent trial was registered at ClinicalTrials.gov (NCT02414399). Before recruitment, written informed consent was sought from caregivers of the participating children in their preferred language. If a caregiver could not read and write, a thumbprint was obtained with a witness countersigning following informed consent.

### Study Design and Sample Collection

We conducted a cross-sectional study using *E coli* isolates derived from samples collected at baseline from children who were enrolled to participate in a randomized controlled trial. A detailed description of the trial’s study design, inclusion criteria, sample collection, and preparations for laboratory processing has been reported [24, 25]. Briefly, between June 2016 and November 2019, children aged 1 to 59 months who were discharged from the hospital in the Kisii and Homa Bay counties of western Kenya were enrolled in a clinical trial testing the efficacy of azithromycin for prevention of morbidity and mortality in the 6 months following hospitalization. Kisii Teaching and Referral Hospital, a level 6 hospital, serves an urban population of about 1.2 million people and is a major referral hospital in western Kenya. Homa Bay County Teaching and Referral Hospital, a level 5 hospital, serves a predominantly rural population of around 1.1 million people. Homa Bay County has one of the highest under-5 childhood mortality rates and HIV prevalence in the country [26].

During enrollment, a physical examination, clinical history, and caregiver interview were conducted. When available, vaccine cards were abstracted into case report forms; when not, a set of questions to ascertain vaccination status against each pathogen was asked and recorded. Fecal samples were collected from children prior to randomization, immediately placed in Cary-Blair media, and sent to the Center for Microbiology Laboratory at the Kenya Medical Research Institute for bacterial culture. The samples were plated on MacConkey agar and incubated aerobically at 37 °C for 24 hours. Distinct morphologies of lactose-fermenting colonies were picked and plated on Mueller-Hilton agar for overnight incubation for biochemical testing. *E coli* isolates were confirmed with the API 20E system (bioMérieux) and reaction to oxidase. The isolates were stored in 15% tryptic soy broth with glycerol prior to resistance testing.

In this nested study, we randomly selected a subset of baseline *E coli* isolates, previously tested for phenotypic resistance [5], for genotypic testing. The selection process utilized simple random sampling with stratification by site. The isolates were suspended in tryptic soy broth (Oxoid) with 15% glycerol and frozen at −80°C. From a random subset of 238 children, baseline *E coli* isolates were shipped on dry ice to the University of Washington *Neisseria* Reference Laboratory for molecular characterization of β-lactamase and macrolide resistance genes. Distinct morphologies of *E coli*, up to 3 in total, were stored at −80°C. Individual morphologies underwent phenotypic antimicrobial susceptibility testing, while DNA extraction took place from the combined vial. Phenotypic resistance was determined by the disc diffusion technique following steps described by the Clinical and Laboratory Standards Institute [27] and detailed in our previous work [5]. Isolates were categorized as ESBL positive if the difference in zone diameters of CTX and CAZ and their combinations with clavulanic acid was >5. DNA extraction was performed on overnight cultures of *E coli* isolates with Qiagen kits. Purified DNA samples were used as templates in polymerase chain reactions with previously published universal primer sets to detect β-lactamase genes (*bla*_CTX-M_, *bla*_TEM_, *bla*_SHV_, *bla*_OXA_, and *bla*_ampC_) [28, 29] and gene-specific primers for detection of macrolide-conferring resistance genes: *erm*(B), *erm*(C), *mef*(A), and *mph*(A) [30, 31]. All polymerase chain reaction assays included negative controls and DNA of characterized positive control strains, including whole genome–sequenced antibiotic-resistant strains obtained from the Antibiotic Resistance Isolate Bank (*E coli* AR 0346, *Klebsiella pneumoniae* AR 0347, *E coli* AR 0348, *K pneumoniae* 0497; Centers for Disease Control and Prevention, Food and Drug Administration) and standard control strains harboring macrolide resistance genes (faculty.washington.edu/marilynr).

### Statistical Analysis

Descriptive statistics were used to characterize variations in social, demographic, and vaccination characteristics between the study sites. The prevalence of each AMR gene, as well as genes grouped by 1 or more β-lactamase– or macrolide resistance–conferring genes, was estimated with 95% CIs calculated by the binomial exact method. CTX-M–type ESBLs are currently the most widely distributed and globally dominant ESBL genotypes [32], while *mph*(A) is the most common genotype for macrolide resistance [30]. Therefore, our outcome variables in correlate analysis, selected a priori, were the presence of CTX-M–type ESBL and *mph*(A) genes detected in *E coli* isolates.

We assessed the following potential correlates in regression analyses: (1) child characteristics (sex, age, study site, HIV exposure, nutritional status, breastfeeding); (2) complete age-appropriate vaccination variables (pneumococcal, rotavirus, DPT [diphtheria, pertussis, and tetanus], and bacille Calmette-Guérin vaccination); (3) hospitalization variables (length of hospital stay and antibiotic use during hospital stay); (4) social-economic variables (caregiver-reported income, caregiver education level); and (5) water, sanitation, and hygiene variables (household toilet type, water source and treatment, and household crowding).

Nutritional status was determined with anthropometric *z* scores constructed per the 2006 World Health Organization growth references for children aged <5 years [33]. We defined stunting as a height-for-age *z* score <−2 SD, underweight as a weight-for-age *z* score <−2 SD, and wasting as weight-for-height/length *z* score <−2 SD. Vaccination status (including date of vaccination) was ascertained from childhood vaccination cards if available at the hospital. If the cards were not available, the caregiver provided a report on the child's vaccination status, including the doses taken and the approximate date. Children were considered fully vaccinated if they received the recommended dosage within 2 weeks of the age specified in the Kenya Vaccine Schedule [34]. In addition, we derived an overall vaccine variable that defined children who had completed all the essential age-appropriate vaccines listed previously (hereinafter, complete age-appropriate vaccination). A household was considered to have access to improved water if the caregiver reported access to reliable piped water in the dwelling or community or if the household primarily used water from a borehole, a protected spring, a well with a pump, bottled water, or rainwater from storage tanks for household chores. Household crowding was defined as >2 individuals sharing a room.

We used a modified Poisson regression model to estimate the relative risk (prevalence ratio) of CTX-M–type ESBL or *mph*(A) detected in *E coli* between children with and without risk factors of interest. Univariable regression models were performed for all predictor variables. In the multivariate regression models, each risk factor, including each vaccine variable, was adjusted for a priori–defined confounders (age, sex, and recruiting site). All statistical analyses were performed in Stata 17 (StataCorp).

## RESULTS

**Figure 1.**
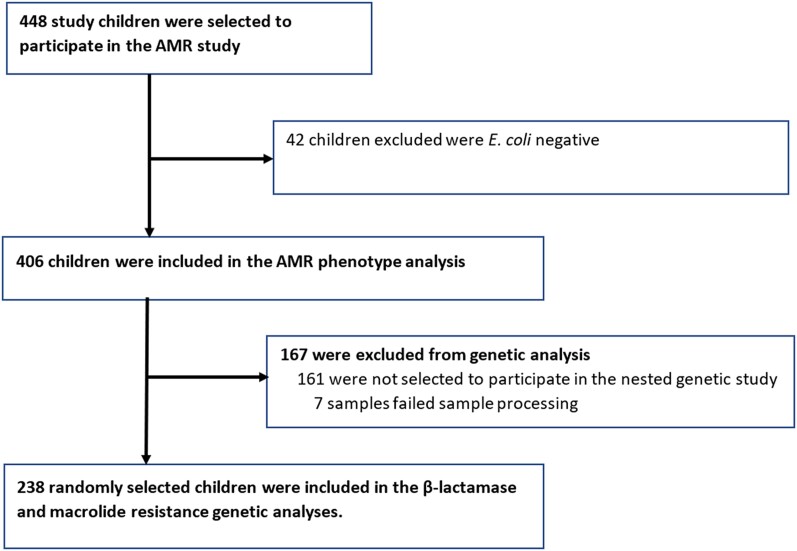
Participant flowchart and reasons for exclusion at each stage. AMR, antimicrobial resistance; *E coli*, *Escherichia coli*.

Of the 1400 children recruited into the parent study, 448 were randomly selected to participate in the AMR phenotypic study and 238 in the AMR genotypic study (Figure 1). Participant characteristics in the parent study and the phenotypic study have been reported elsewhere [5, 25].

### Population Characteristics

The median age of children in the AMR genetic study was 19 months (IQR, 9–32), and 90 (37.8%) were female. The prevalence of exclusive breastfeeding was higher among children recruited at the Homa Bay site (61.7%) than the Kisii site (31.9%). Similarly, HIV seropositivity was higher among children recruited in the Homa Bay site (25.5%) vs the Kisii site (6.2%). Overall, the median hospital stay was 3 days (IQR, 2–5) in Kisii and 3 days (IQR, 2–5) in the Homa Bay site. Antibiotic use during hospitalization was 84.8%, with marked heterogeneity between sites (Table 1). In addition, geographic variability in antibiotic administration during hospitalization was evident in the prescription of gentamicin and penicillin. Furthermore, we observed marked variations in complete age-appropriate vaccination (51.4% vs 23.4%), which was largely driven by rotavirus vaccination (81.9% vs 69.1%) and measles vaccination (71.5% vs 45.7%) at the Kisii and Homa Bay sites, respectively (Supplementary Figure 1).

**Table 1.** Characteristics of Children Enrolled in the Genetic Antimicrobial Resistance Study

	No. (%)^a^		
	Homa Bay (n = 94)	Kisii (n = 144)	Total (n = 238)
Child characteristics			
Age, mo			
0–5	8 (9)	19 (13)	27 (11)
6–11	14 (15)	31 (22)	45 (19)
12–23	31 (33)	42 (29)	73 (31)
24–59	41 (44)	52 (36)	93 (39)
Sex			
Male	58 (62)	90 (63)	148 (62)
Female	36 (38)	54 (38)	90 (38)
Breastfeeding^b^			
Exclusively breastfed	58 (62)	46 (32)	104 (44)
Partially breastfed	35 (37)	82 (57)	117 (49)
Unknown	1 (1)	16 (11)	17 (7)
Child HIV status^c^			
HIV unexposed	69 (74)	130 (94)	199 (86)
HIV positive or exposed	24 (26)	9 (6)	33 (14)
Underweight (WAZ <−2)			
WAZ ≥−2	86 (91)	124 (86)	210 (88)
WAZ <−2	8 (9)	20 (14)	28 (12)
Stunting (HAZ/LAZ <−2)			
HAZ/LAZ ≥−2	70 (75)	103 (72)	173 (73)
HAZ/LAZ <−2	23 (25)	40 (28)	63 (27)
Acute malnutrition			
None	83 (88)	128 (89)	211 (89)
MAM	8 (9)	7 (5)	15 (6)
SAM	3 (3)	9 (6)	12 (5)
Vaccination status			
Pneumococcal vaccination^d^			
No	5 (5)	13 (9)	18 (8)
Yes	89 (95)	131 (91)	220 (92)
Rotavirus vaccination^e^			
No	29 (31)	26 (18)	55 (23)
Yes	65 (69)	118 (82)	183 (77)
DTP vaccination^f^			
No	7 (7)	9 (6)	16 (7)
Yes	87 (93)	135 (94)	222 (93)
Measles vaccination^g^			
No	51 (54)	41 (28)	92 (39)
Yes	43 (46)	103 (72)	146 (61)
Completed all essential vaccines^h^			
No	72 (77)	70 (49)	142 (60)
Yes	22 (23)	74 (51)	96 (40)
Hospitalization information			
Length of hospital stay			
≤3 d	50 (53)	75 (53)	125 (53)
>3 d	44 (47)	67 (47)	111 (47)
Antibiotic use during admission			
No	25 (27)	11 (8)	36 (15)
Yes	69 (73)	133 (92)	202 (85)
Ceftriaxone use during admission			
No	57 (61)	102 (71)	159 (67)
Yes	37 (39)	42 (29)	79 (33)
Gentamicin use during admission			
No	65 (69)	47 (33)	112 (47)
Yes	29 (31)	97 (67)	126 (53)
Chloramphenicol use during admission			
No	92 (98)	135 (94)	227 (95)
Yes	2 (2)	9 (6)	11 (5)
Penicillin use during admission			
No	61 (65)	38 (26)	99 (42)
Yes	33 (35)	106 (74)	139 (58)
Household information			
Caregiver-reported income, Kenyan shilling			
≥5000	13 (14)	53 (37)	66 (28)
<5000	74 (79)	86 (60)	160 (67)
Unknown or refuse to answer	7 (7)	5 (3)	12 (5)
Crowding (>2 persons per room)			
No (≤2)	32 (34)	89 (62)	121 (51)
Yes (>2)	62 (66)	55 (38)	117 (49)
Improved water source			
No	23 (24)	18 (13)	41 (17)
Yes	71 (76)	126 (88)	197 (83)
Treated drinking water			
No	26 (28)	89 (63)	115 (49)
Yes	67 (72)	53 (37)	120 (51)
Toilet			
Private, for household only	30 (33)	80 (56)	110 (47)
Shared with ≥1 household	50 (54)	64 (44)	114 (48)
Open defecation	12 (13)	0 (0)	12 (5)

Abbreviations: DPT, diphtheria, pertussis, and tetanus; HAZ, height for age; LAZ, length for age; MAM, moderate acute malnutrition; SAM, severe acute malnutrition; WAZ, weight for age.

^a^Column percentages.

^b^Whether the child is currently breastfeeding (≤6 months old) or if the caregiver practiced breastfeeding when the child was ≤6 months old.

^c^Six children had exposure with an unknown infection status and were excluded.

^d^Pneumococcal vaccination defined as vaccination completed for the 6-, 10-, and 14-week schedule or up to the age of the child allowing a 2-week margin.

^e^Rotavirus vaccine defined as vaccination completed for the 6- and 10-week schedule or up to the age of the child allowing a 2-week margin.

^f^DPT vaccine defined as vaccination completed for the 6-, 10-, and 14-week schedule or up to the age of the child allowing a 2-week margin.

^g^Measles vaccine defined as vaccination completed for the 9- and 18-month schedule or up to the age of the child allowing a 2-week margin.

^h^All essential vaccination defined as having complete vaccination for pneumococcal, rotavirus, DPT, measles, and bacille Calmette-Guérin.

### Distribution of Genetic and Phenotypic AMR Among Study Participants

Overall, 89.9% of children had at least 1 β-lactamase–conferring gene, and 47.1% had at least 1 macrolide resistance–conferring gene. The most common β-lactamase–conferring gene was *bla*_TEM_ (87.9%; 95% CI, 83.0%–91.7%), followed by *bla*_SHV_ (48.1%; 95% CI, 41.6%–54.7%), *bla*_CTX-M_ (38.1%; 95% CI, 31.9%–44.6%), *bla*_ampC_ (15.9%; 95% CI, 11.5%–21.2%), and *bla*_OXA_ (10.5%; 95% CI, 6.9%–15.1%). The most common macrolide-resistance conferring gene was *mph*(A) (45.6%; 95% CI, 39.2%–52.2%), followed by *erm*(B) (1.67%; 95% CI, .46%–4.23%), *mef*(A) (0.84%; 95% CI, .10%–2.99%), and *erm*(C) (0%; 95% CI, 0%–1.53%). Detailed site variations in prevalence of β-lactamase genes and macrolide resistance genes are presented in Supplementary Figure 2. The subsequent analyses examine the risk factors of *bla*_CTX-M_ as a proxy for ESBL-producing genes and *mph*(A) as a proxy for macrolide resistance. Among the 214 children with at least 1 ESBL-conferring gene, the presence of phenotypic ESBL was 47.7%, and among those with a macrolide-conferring gene (n = 112), the prevalence of azithromycin resistance was 63.4%. Sixty-one (25.6%) children had cocarriage of *bla*_CTX-M_ and *mph*(A) genes. Further details on phenotypic ESBL among children whose *E coli* isolates underwent genetic analyses are shown in Supplementary Figures 3 and 4.

### Risk Factors for *bla*_CTX-M_ Carriage

In the univariable and multivariable analyses, children who had received the age-appropriate pneumococcal vaccine doses in full had >40% lower likelihood of *bla*_CTX-M_ detection in their *E coli* isolates when compared with children who did not (adjusted prevalence ratio [aPR], 0.57; 95% CI, .38–.85; *P* = .005). However, there were no significant associations between *bla*_CTX-M_ in *E coli* and full age-appropriate rotavirus vaccination (aPR, 0.82; 95% CI, .56–1.21; *P* = .319), DPT vaccination (aPR, 0.70; 95% CI, .42–1.12; *P* = .137), or measles vaccination (aPR, 1.06; 95% CI, .73–1.54; *P* = .746). Antibiotic use during hospital admission was associated with a higher likelihood of harboring *bla*_CTX-M_–producing *E coli* (aPR, 2.47; 95% CI, 1.12–5.43; *P* = .025), and this appeared to be driven by children who received ceftriaxone during admission as compared with other antibiotics (aPR, 2.51; 95% CI, 1.79–3.52; *P*< .001). Administration of gentamicin (aPR, 0.62; 95% CI, .44–.87; *P* = .006) and penicillin (aPR, 0.63; 95% CI, .45–.88; *P* = .008) was associated with a lower likelihood of harboring *bla*_CTX-M_–producing *E coli* when compared with other antibiotics. Relative to children who stayed in the hospital for ≤3 days, longer hospital stays (>3 days) were associated with an increased risk of carrying *bla*_CTX-M_–producing *E coli* (aPR, 1.42; 95% CI, 1.00–2.01; *P* = .052). In addition, there was an increased risk of *bla*_CTX-M_–producing *E coli* among children residing in households that practiced open defecation (aPR, 2.47; 95% CI, 1.40–4.36; *P* = .002). Site, age, sex, improved water sources, caregiver income, and anthropometric measures were not significantly associated with *bla*_CTX-M_–producing *E coli* (Table 2).

**Table 2.** Risk Factors of CTX-M in Commensal *Escherichia coli* Isolated From Fecal Samples of the Participating Children

	No. (%)^a^		Model 1: Univariable Analysis			Model 2: Multivariable Analysis		
	CTX-M+ (n = 91)	CTX-M– (n = 147)	PR	95% CI	*P* Value	Adjusted PR^b^	95% CI	*P* Value
Location of the facility						…	…	…
Kisii	58 (64)	86 (59)	1 [Ref]					
Homa Bay	33 (36)	61 (41)	0.87	.62–1.22	.428	0.89	.63- 1.25	.504
Child characteristics								
Age, mo						…	…	…
0–5	13 (14)	14 (10)	1 [Ref]					
6–11	18 (20)	27 (18)	0.83	.49–1.41	.494	0.84	.49- 1.43	.516
12–23	28 (31)	45 (31)	0.80	.49–1.30	.362	0.81	.49- 1.33	.410
24–59	32 (35)	61 (41)	0.71	.44–1.16	.172	0.73	.45- 1.18	.202
Sex						…	…	…
Male	55 (60)	93 (63)	1 [Ref]					
Female	36 (40)	54 (37)	1.08	.77–1.50	.661	1.08	.78- 1.50	.647
Breastfeeding^c^								
Exclusively breastfed	39 (43)	65 (44)	1 [Ref]			1 [Ref]		
Partially breastfed	46 (51)	71 (48)	1.05	.75–1.47	.782	1.01	.72–1.42	.949
Unknown	6 (7)	11 (7)	0.94	.47–1.88	.864	0.84	.41–1.75	.646
Child HIV status^d^								
HIV unexposed	80 (89)	119 (84)	1 [Ref]			1 [Ref]		
HIV positive or exposed	10 (11)	23 (16)	0.75	.44–1.30	.310	0.79	.45–1.38	.413
Underweight (WAZ <−2)								
WAZ ≥−2	77 (85)	133 (90)	1 [Ref]			1 [Ref]		
WAZ <−2	14 (15)	14 (10)	1.36	.90–2.06	.140	1.35	.89–2.05	.157
Stunting (HAZ/LAZ <−2)								
HAZ/LAZ ≥−2	66 (73)	107 (73)	1 [Ref]			1 [Ref]		
HAZ/LAZ <−2	24 (27)	39 (27)	1.00	.69–1.44	.994	1.02	.70–1.49	.904
Acute malnutrition								
None	78 (86)	133 (90)	1 [Ref]			1 [Ref]		
MAM	7 (8)	8 (5)	1.26	.71–2.23	.423	1.30	.73–2.33	.375
SAM	6 (7)	6 (4)	1.35	.75–2.45	.319	1.30	.70–2.40	.409
Vaccination status								
Pneumococcal vaccination^e^								
No	12 (13)	6 (4)	1 [Ref]			1 [Ref]		
Yes	79 (87)	141 (96)	0.54	.37–.78	.001	0.57	.38–.85	.005
Rotavirus vaccination^f^								
No	23 (25)	32 (22)	1 [Ref]			1 [Ref]		
Yes	68 (75)	115 (78)	0.89	.62–1.28	.526	0.82	.56–1.21	.319
DPT vaccination^g^								
No	9 (10)	7 (5)	1 [Ref]			1 [Ref]		
Yes	82 (90)	140 (95)	0.66	.41–1.05	.077	0.70	.43–1.12	.137
Measles vaccination^h^								
No	32 (35)	60 (41)	1 [Ref]					
Yes	59 (65)	87 (59)	1.16	.82–1.64	.391	1.06	.73–1.54	.746
Completed all essential vaccines^i^								
No	56 (62)	86 (59)	1 [Ref]			1 [Ref]		
Yes	35 (38)	61 (41)	0.92	.66–1.29	.645	0.85	.60–1.20	.356
Hospitalization information								
Length of hospital stay								
≤3 d	39 (44)	86 (59)	1 [Ref]			1 [Ref]		
>3 d	50 (56)	61 (41)	1.44	1.04–2.01	.030	1.42	1.00–2.01	.052
Any antibiotic use during admission								
No	6 (7)	30 (20)	1 [Ref]			1 [Ref]		
Yes	85 (93)	117 (80)	2.52	1.19–5.34	.015	2.47	1.12–5.43	.025
Ceftriaxone use during admission^j^								
No	34 (40)	89 (76)	1 [Ref]			1 [Ref]		
Yes	51 (60)	28 (24)	2.34	1.68–3.25	<.001	2.51	1.79–3.52	<.001
Gentamicin use during admission^j^								
No	41 (48)	35 (30)	1 [Ref]			1 [Ref]		
Yes	44 (52)	82 (70)	0.65	.47–.89	.007	0.62	.44–.87	.001
Chloramphenicol use during admission^j^								
No	83 (98)	108 (92)	1 [Ref]			1 [Ref]		
Yes	2 (2)	9 (8)	0.42	.12–1.49	.178	0.44	.12–1.58	.207
Penicillin use during admission^j^								
No	35 (41)	28 (25)	1 [Ref]			1 [Ref]		
Yes	50 (59)	89 (75)	0.65	.47–.89	.007	0.63	.45–.88	.008
Household information								
Caregiver-reported income, Kenyan shilling								
≥5000	30 (33)	36 (24)	1 [Ref]			1 [Ref]		
<5000	58 (64)	102 (69)	0.80	.57–1.12	.186	0.78	.55–1.10	.156
Unknown or refuse to answer	3 (3)	9 (6)	0.55	.20–1.52	.249	0.53	.18–1.53	.242
Crowding (>2 persons per room)								
No (≤2)	46 (51)	75 (51)	1 [Ref]			1 [Ref]		
Yes (>2)	45 (49)	72 (49)	1.01	.73–1.40	.944	1.05	.75–1.48	.762
Improved water source								
No	12 (13)	29 (20)	1 [Ref]			1 [Ref]		
Yes	79 (87)	118 (80)	1.37	.83–2.27	.223	1.33	.79–2.24	.279
Treated drinking water								
No	46 (51)	69 (48)	1 [Ref]			1 [Ref]		
Yes	44 (49)	76 (52)	0.92	.66–1.27	.600	0.97	.68–1.37	.854
Toilet								
Private, for household only	37 (41)	73 (50)	1 [Ref]			1 [Ref]		
Shared with ≥1 household	46 (51)	68 (47)	1.20	.85–1.69	.302	1.22	.86–1.74	.267
Open defecation	8 (9)	4 (3)	1.98	1.23–3.20	.005	2.47	1.40–4.36	.002

Model 1 presents results from the univariable Poisson regression model with robust SE, and model 2 presents results of an adjusted multivariable Poisson regression model (adjusted for age, sex, and site) with robust SE. CTX-M+ and CTX-M– denote the presence and absence of the CTX-M gene in the isolated *E coli* from fecal samples.

Abbreviations: DPT, diphtheria, pertussis, and tetanus; HAZ, height for age; LAZ, length for age; MAM, moderate acute malnutrition; PR, prevalence ratio; Ref, reference; SAM, severe acute malnutrition; WAZ, weight for age.

^a^Column percentages.

^b^Adjusted for sex, age, and site. Models including one of the three confounders were adjusted for the other two.

^c^Whether the child is currently breastfeeding (≤6 months old) or if the mother practiced breastfeeding when the child was ≤6 months old.

^d^Six children had exposure with an unknown infection status and were excluded.

^e^Pneumococcal vaccination defined as vaccination completed for the 6-, 10-, and 14-week schedule or up to the age of the child allowing a 2-week margin.

^f^Rotavirus vaccine defined as vaccination completed for the 6- and 10-week schedule or up to the age of the child allowing a 2-week margin.

^g^DPT vaccine defined as vaccination completed for the 6-, 10-, and 14-week schedule or up to the age of the child allowing a 2-week margin.

^h^Measles vaccine defined as vaccination completed for the 9- and 18-month schedule or up to the age of the child allowing a 2-week margin.

^i^All essential vaccination defined as having complete vaccination for pneumococcal, rotavirus, DPT, measles, and bacille Calmette-Guérin.

^j^Among children who received at least 1 antibiotic during hospitalization (antibiotic of interest vs other antibiotics).

### Risk Factors for *mph*(A) Carriage

Children who had completed all age-appropriate essential vaccination had a 32% decrease in risk of harboring *mph*(A)–positive *E coli* (aPR, 0.68; 95% CI, .49–.93; *P* = .017). In particular, full age-appropriate measles vaccination was associated with a 30% decrease in *mph*(A)–producing *E coli* (aPR, 0.70; 95% CI, .52–.95; *P* = .022). However, there were no associations between detection of *mph*(A)–producing *E coli* and pneumococcal, rotavirus, or DPT vaccination (Table 3).

**Table 3.** Risk Factors of the *mph*(A) Gene in Commensal *Escherichia coli* Isolated From Fecal Samples of the Participating Children

	No. (%)^a^		Model 1: Univariable Analysis			Model 2: Multivariable Analysis		
	*mph*(A)+ (n = 109)	*mph*(A)– (n = 129)	PR	95% CI	*P* Value	Adjusted PR^b^	95% CI	*P* Value
Location of the facility						…	…	…
Kisii	64 (59)	80 (62)	1 [Ref]					
Homa Bay	45 (41)	49 (38)	1.08	.81–1.42	.602	1.09	.82- 1.44	.560
Child characteristics								
Age, mo						…	…	…
0–5	14 (13)	13 (10)	1 [Ref]					
6–11	21 (19)	24 (19)	0.90	.56–1.45	.667	0.92	.57- 1.48	.719
12–23	28 (26)	45 (35)	0.74	.46–1.18	.205	0.74	.46- 1.18	.207
24–59	46 (42)	47 (36)	0.95	.63–1.45	.825	0.94	.62- 1.43	.777
Sex						…	…	…
Male	61 (56)	87 (67)	1 [Ref]					
Female	48 (44)	42 (33)	1.29	.98–1.70	.065	1.28	.98- 1.69	.075
Breastfeeding^c^								
Exclusively breastfed	53 (49)	51 (40)	1 [Ref]			1 [Ref]		
Partially breastfed	49 (45)	68 (53)	0.82	.62–1.09	.178	0.82	.61–1.10	.177
Unknown	7 (6)	10 (8)	0.81	.44–1.47	.486	0.82	.44–1.54	.540
Child HIV status^d^								
HIV unexposed	95 (88)	104 (84)	1 [Ref]			1 [Ref]		
HIV positive or exposed	13 (12)	20 (16)	0.83	.53–1.29	.401	0.80	.51–1.26	.343
Underweight (WAZ <−2)								
WAZ ≥−2	99 (91)	111 (86)	1 [Ref]			1 [Ref]		
WAZ <−2	10 (9)	18 (14)	0.76	.45–1.27	.294	0.82	.48–1.38	.451
Stunting (HAZ/LAZ <−2)								
HAZ/LAZ ≥−2	79 (73)	94 (73)	1 [Ref]			1 [Ref]		
HAZ/LAZ <−2	29 (27)	34 (27)	1.01	.74–1.38	.960	1.08	.79–1.48	.622
Acute malnutrition								
None	96 (88)	115 (89)	1 [Ref]			1 [Ref]		
MAM	8 (7)	7 (5)	1.17	.71–1.93	.531	1.16	.70–1.90	.569
SAM	5 (5)	7 (5)	0.92	.46–1.82	.802	0.89	.44–1.82	.753
Vaccination status								
Pneumococcal vaccination^e^								
No	8 (7)	10 (8)	1 [Ref]			1 [Ref]		
Yes	101 (93)	119 (92)	1.03	.60–1.77	.906	1.03	.59–1.78	.926
Rotavirus vaccination^f^								
No	25 (23)	30 (23)	1 [Ref]			1 [Ref]		
Yes	84 (77)	99 (77)	1.01	.73–1.40	.954	1.06	.75–1.50	.725
DPT vaccination^g^								
No	6 (6)	10 (8)	1 [Ref]			1 [Ref]		
Yes	103 (94)	119 (92)	1.24	.65–2.37	.521	1.30	.69–2.47	.421
Measles vaccination^h^								
No	50 (46)	42 (33)	1 [Ref]					
Yes	59 (54)	87 (67)	0.74	.57–.98	.033	0.70	.52–.95	.022
Completed all essential vaccines^i^								
No	74 (68)	68 (53)	1 [Ref]			1 [Ref]		
Yes	35 (32)	61 (47)	0.70	.51–.95	.023	0.68	.49–.93	.017
Hospitalization information								
Length of hospital stay								
≤3 d	47 (44)	78 (60)	1 [Ref]			1 [Ref]		
>3 d	60 (56)	51 (40)	1.44	1.08–1.91	.012	1.47	1.10–1.98	.009
Antibiotic use during admission								
No	10 (9)	26 (20)	1 [Ref]			1 [Ref]		
Yes	99 (91)	103 (80)	1.76	1.02–3.05	.042	1.83	1.06–3.18	.031
Ceftriaxone use during admission^j^								
No	59 (60)	64 (62)	1 [Ref]			1 [Ref]		
Yes	40 (40)	39 (38)	1.06	.79–1.40	.711	1.00	.74–1.34	.981
Gentamicin use during admission^j^								
No	33 (33)	43(42)	1 [Ref]			1 [Ref]		
Yes	66 (67)	60 (58)	1.21	.89–1.64	.231	1.33	.97–1.83	.073
Chloramphenicol use during admission^j^								
No	94 (95)	97 (94)	1 [Ref]			1 [Ref]		
Yes	5 (5)	6 (6)	0.92	.48–1.80	.815	1.04	.53–2.01	.916
Penicillin use during admission^j^								
No	27 (27)	36 (35)	1 [Ref]			1 [Ref]		
Yes	72 (73)	67 (65)	1.21	.87–1.68	.257	1.36	.97–1.89	.071
Household characteristics								
Caregiver-reported monthly income, Kenyan shilling								
≥5000	33 (30)	33 (26)	1 [Ref]			1 [Ref]		
<5000	73 (67)	87 (67)	0.91	.68–1.23	.543	0.89	.65–1.20	.438
Unknown or refuse to answer	3 (3)	9 (7)	0.50	.18–1.37	.179	0.42	.15–1.22	.110
Crowding (>2 persons per room)								
No (≤2)	53 (49)	68 (53)	1 [Ref]			1 [Ref]		
Yes (>2)	56 (51)	61 (47)	1.09	.83–1.44	.531	1.05	.79–1.41	.717
Improved water source								
No	19 (17)	22 (17)	1 [Ref]			1 [Ref]		
Yes	90 (83)	107 (83)	0.99	.69–1.42	.939	1.04	.72–1.49	.851
Treated drinking water								
No	49 (46)	66 (52)	1 [Ref]			1 [Ref]		
Yes	58 (54)	62 (48)	1.13	0.86–1.50	.381	1.12	.83–1.51	.456
Toilet								
Private, for household only	46 (43)	64 (50)	1 [Ref]			1 [Ref]		
Shared with ≥1 households	53 (50)	61 (47)	1.11	0.83–1.50	.483	1.15	.85–1.56	.368
Open defecation	8 (7)	4 (3)	1.59	1.01–2.52	.046	1.58	.95–2.62	.076

Model 1 presents results from the univariable Poisson regression model with robust SE, and model 2 presents results of an adjusted multivariable Poisson regression model (adjusted for age, sex, and site) with robust SE. *mph*(A)+ and *mph*(A)– denote the presence and absence of the *mph*(A) gene in the isolated *E coli* from fecal samples.

Abbreviations: DPT, diphtheria, pertussis, and tetanus; HAZ, height for age; LAZ, length for age; MAM, moderate acute malnutrition; PR, prevalence ratio; Ref, reference; SAM, severe acute malnutrition; WAZ, weight for age.

^a^Column percentages.

^b^Adjusted for sex, age, and site. Models including one of the three confounders were adjusted for the other two.

^c^Whether the child is currently breastfeeding (≤6 months old) or if the mother practiced breastfeeding when the child was ≤6 months old.

^d^Six children had exposure with an unknown infection status and were excluded.

^e^Pneumococcal vaccination defined as vaccination completed for the 6-, 10-, and 14-week schedule or up to the age of the child allowing a 2-week margin.

^f^Rotavirus vaccine defined as vaccination completed for the 6- and 10-week schedule or up to the age of the child allowing a 2-week margin.

^g^DPT vaccine defined as vaccination completed for the 6-, 10-, and 14-week schedule or up to the age of the child allowing a 2-week margin.

^h^Measles vaccine defined as vaccination completed for the 9- and 18-month schedule or up to the age of the child allowing a 2-week margin.

^i^All essential vaccination defined as having complete vaccination for pneumococcal, rotavirus, DPT, measles, and bacille Calmette-Guérin.

^j^Among children who received at least 1 antibiotic during hospitalization (antibiotic of interest vs other antibiotics).

As observed with *bla*_CTX-M_, antibiotic use during hospital admission was associated with an increased risk of harboring *mph*(A)–positive *E coli* (aPR, 1.83; 95% CI, 1.06–3.18; *P* = .031). Length of hospital stay and residing in households that practiced open defecation were also independent risk factors for *mph*(A) (Table 3).

## DISCUSSION

Children discharged from hospitals in SSA are at increased risk of morbidity and mortality as compared with children in their communities [9]. Here we describe patterns of β-lactamase– and macrolide resistance–conferring genes among children discharged from the hospital: a population at high risk for morbidity and mortality, as well as one that may serve as a reservoir for the transmission of AMR bacteria within households and communities after children return home. First, our results demonstrate that β-lactamase– and macrolide resistance–conferring genes are common in *E coli* isolates from children at the point of hospital discharge in western Kenya, for which the clinical and transmission implications are yet to be defined. Second, our results indicate that, in addition to factors related to hospitalization, age-appropriate vaccination against common infectious diseases and access to sanitation were associated with the likelihood of resistant gene carriage. Strategies to reduce antibiotic resistance should focus not only on antibiotic stewardship programs and infection control in hospitals but also on sanitation improvements within households and communities, as well as efforts to increase routine vaccination coverage.

β-Lactamase– and macrolide resistance–conferring genes were common in *E coli* isolates from children at hospital discharge. Due to the timing of sample collection (hospital discharge), we cannot determine whether resistance was present prior to admission or acquired during hospitalization. The presence of CTX-M–harboring *E coli* in children discharged from the hospital is a public health concern, as (1) the mobile genetic elements carrying resistance genes may be transmitted to other pathogenic bacteria and (2) resistant bacteria may be transmitted within households and in the broader community. Studies conducted across SSA and elsewhere have demonstrated a high prevalence of *bla*_CTX-M_, *bla*_TEM_, and *bla*_SHV_ [20, 35–37], ranging from 22% to 96% in samples from patients at health facilities, consistent with our findings.

Our findings are consistent with results from controlled experiments that have documented a strong protective effect of vaccination against AMR [38, 39]. Vaccines can protect against AMR by reducing antibiotic-treated illnesses or by reducing circulation of resistant pathogens [38]. For example, the introduction of pneumococcal vaccination has decreased not only antibiotic use [40] but also the prevalence of AMR infections in all age groups [16, 41, 42]. Similarly, the introduction of influenza vaccination has been associated with a reduction in secondary bacterial infections, leading to a reduction in antibiotic prescriptions and AMR [18]. We did not observe an association between rotavirus vaccination and AMR. This may be due to the fact that rotavirus primarily affects infants, and our study population included children up to 5 years of age, in whom the benefits of rotavirus vaccination on AMR may be attenuated. In addition, the comparison groups for vaccination were children who were partially vaccinated since there were no nonvaccinated children, which may partially account for the lack of significant effects observed.

As reported in our phenotypic analyses [5], most admitted children received antibiotic treatment during hospitalization. We observed a significant relationship between *bla_C_*_TX-M_ and *mph*(A) genes and inpatient antibiotic use, suggesting selection for antibiotic-resistant bacteria during hospitalization. Use of the third-generation cephalosporin ceftriaxone during the hospital stay was particularly predictive of *bla*_CTX-M_ positivity, while use of gentamicin and penicillin was significantly associated with a lower risk of *bla*_CTX-M_ positivity, likely due to ceftriaxone exposure in the comparison group. We observed a significant relationship between AMR genes and length of hospital stay, reflecting either increasing exposure to nosocomial AMR bacteria or more prolonged antibiotic exposure consistent with previous findings [6].

Surprisingly, we did not find any association between *bla*_CTX-M_ or *mph*(A), and some participants' sociodemographic factors. The findings contrast with previous findings that showed a significant association between AMR and age [6, 43], household crowding [43], HIV infection/exposure, and acute malnutrition [43]. Although interventions targeting water, sanitation, and hygiene have been shown to reduce AMR [44], the independent effect of improved access to a safe water supply or to treated water remains controversial [44, 45]. We observed a significant association between CTX-M–type ESBL and the practice of open defecation, consistent with a recent multicounty analysis [44] and our previous phenotypic analysis [5]. These results establish that although the hospital environment remains an important reservoir of AMR bacteria [6], there is considerable community acquisition of AMR that may be mitigated by improvements in sanitation. At the hospital level, the improvement of sanitation and infection control practices is essential.

Our study has important strengths. First, there are few studies that provide a comprehensive assessment of the patient-level impact of routine vaccination on AMR among children discharged from the hospital who are at a high risk of morbidity and mortality. Second, most studies that assessed the impact of vaccines on AMR were conducted in high-income countries where the burden of infectious diseases is relatively low. Our study was conducted in a periurban setting in western Kenya and therefore provides important data on AMR from a low-income country with a high burden of infectious diseases.

A limitation to our study is that the recruitment of children discharged from the hospital excluded those who died during hospitalization, a population potentially at the highest risk of AMR carriage. Therefore, the burden of AMR-conferring genes in *E coli* that we observed is likely an underestimate of the true burden among hospitalized children. In addition, we did not collect stool samples at hospital admission; thus, we cannot determine whether AMR was acquired during hospitalization or prior to admission. Hospital exposure could have obscured the importance of community-based factors, such as water sources, given that hospital exposure is more proximal. Furthermore, the study was conducted in 2 counties of rural western Kenya and, as such, is difficult to generalize to other settings. Finally, we cannot exclude the possibility that the identified risk factors, including the lack of age-appropriate vaccination and open defecation, may be explained by confounders not measured in this study.

## CONCLUSION

Addressing AMR is a global health priority, and identifying risk factors for AMR in a population for whom antibiotics are likely necessary and potentially lifesaving can inform interventions for reducing AMR. The World Health Organization acknowledges the limited evidence on the impact of existing vaccines on AMR and recommends further research to develop a robust evidence base [19]. Our study provides evidence that routine vaccination and basic sanitation practices are associated with lower levels of AMR carriage. If this is confirmed in other studies, AMR control programs in similar settings should consider the scale-up of routine immunization, basic sanitation, and hospital infection control in addition to antimicrobial stewardship, as a strategy to combat the increasing burden of AMR. Further development of novel vaccines for childhood infections may also play an important complementary role in mitigating the increasing burden of AMR.

## Supplementary Material

ofae307_Supplementary_Data

## Supplementary Data

Supplementary materials are available at *Open Forum Infectious Diseases* online. Consisting of data provided by the authors to benefit the reader, the posted materials are not copyedited and are the sole responsibility of the authors, so questions or comments should be addressed to the corresponding author.

## Notes

***Author contributions*.** Conceptualization and design: O. O. S., S. N. T., B. O. S., S. K., F. C. F., J. L. W., P. B. P. Performed the experiments: O. O. S., S. N. T., R. P., E. W., D. R., K. K., B. O. S., P. B. P. Data curation: P. M., K. D. T., M. M. D. Funding acquisition: J. L. W., P. B. P. Project administration and investigation: O. O. S., K. D. T., S. N. T., M. M. D., D. R., K. K., B. O. S., S. K., P. B. P. Formal data analysis: P. M. Writing–original draft: P. M. Writing–review and editing: P. M., O. O. S., K. D. T., S. N. T., R. P., E. W., M. M. D., D. R., K. K., B. O. S., S. K., F. C. F., J. L. W., P. B. P. All authors have read, and confirm that they meet, ICMJE criteria for authorship.

***Data availability statement*.** All data files are available from Harvard Dataverse (https://doi.org/10.7910/DVN/CEJVKZ).

***Disclaimer.*** The funders had no role in study design, data collection and analysis, decision to publish, or preparation of the manuscript.

***Financial support***. This work was supported by the Eunice Kennedy Shriver National Institute of Child Health and Human Development (National Institutes of Health award R01HD079695 to J. L. W.); and the University of Washington Royalty Research Fund (#A138206 awarded to P. B. P.).
