# Supplementary material for: β-Lactamase and Macrolide Resistance Gene Carriage in Escherichia coli Isolates Among Children Discharged From Inpatient Care in Western Kenya: A Cross-sectional Study
Source: Open Forum Infect Dis. 2024 Jun 3;11(6):ofae307. doi: 10.1093/ofid/ofae307 (PMC11210497; doi:10.1093/ofid/ofae307)
Supplement: ofae307_Supplementary_Data [file ofae307_supplementary_data.docx]

Supplementary material


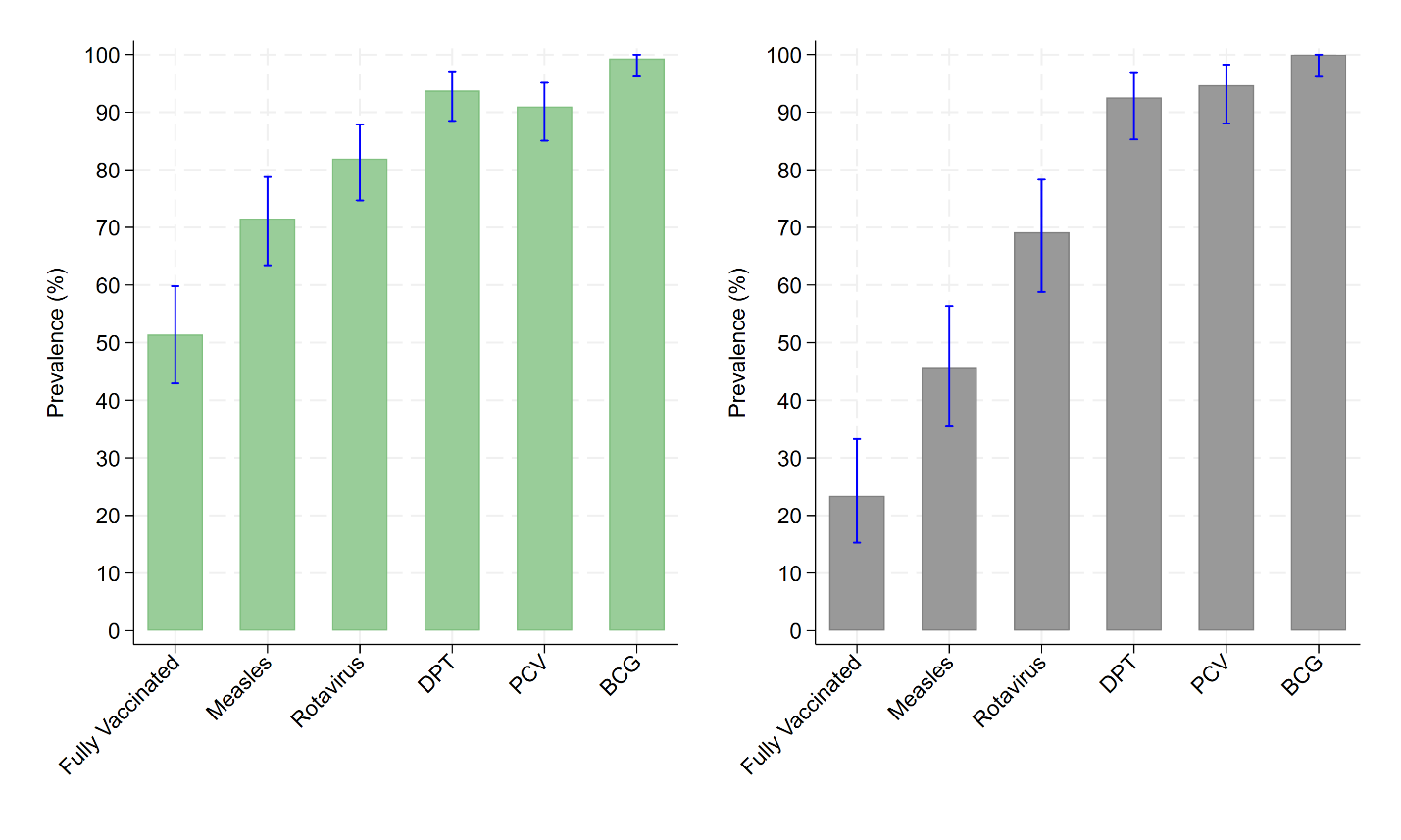


**B**

**A**

**Supplementary Figure 1:** Prevalence of age-adjusted complete vaccine uptake among children admitted in the health facilities in Kisii (Panel A) and Homa Bay (Panel B) hospital study sites. Fully Vaccinated means that the child had completed the required dosage for their age for the following vaccines: Measles, rotavirus, DPT, PCV and BCG. The error bars represent the exact 95% confidence intervals.


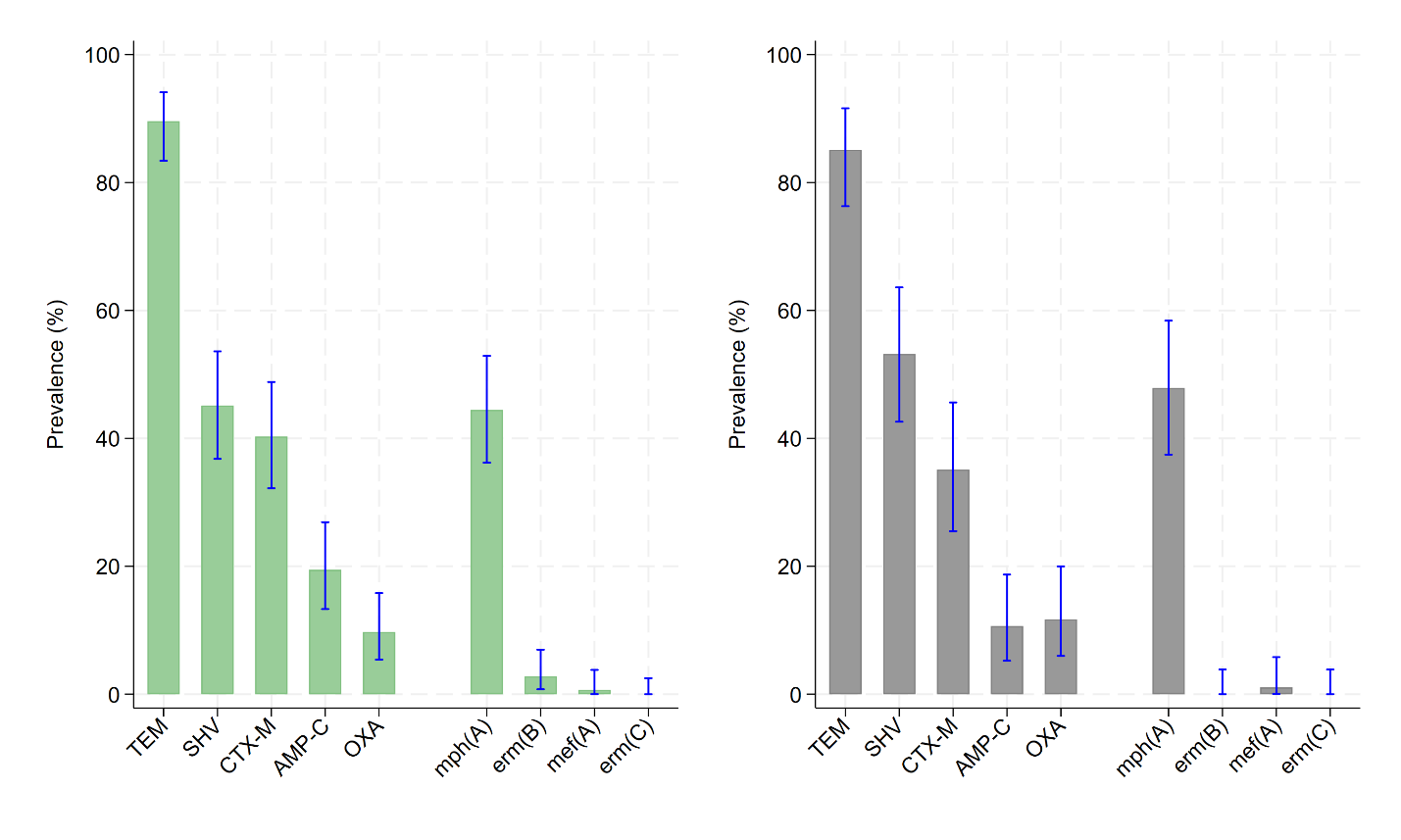


**B**

**A**

**Supplementary Figure 2:** β-lactamase and macrolide resistance genes detected in samples collected from children at the point of discharge from hospital segregated by site. Panels A and B represent children admitted to Kisii and Homa Bay site respectively. The error bars represent the exact 95% confidence intervals.


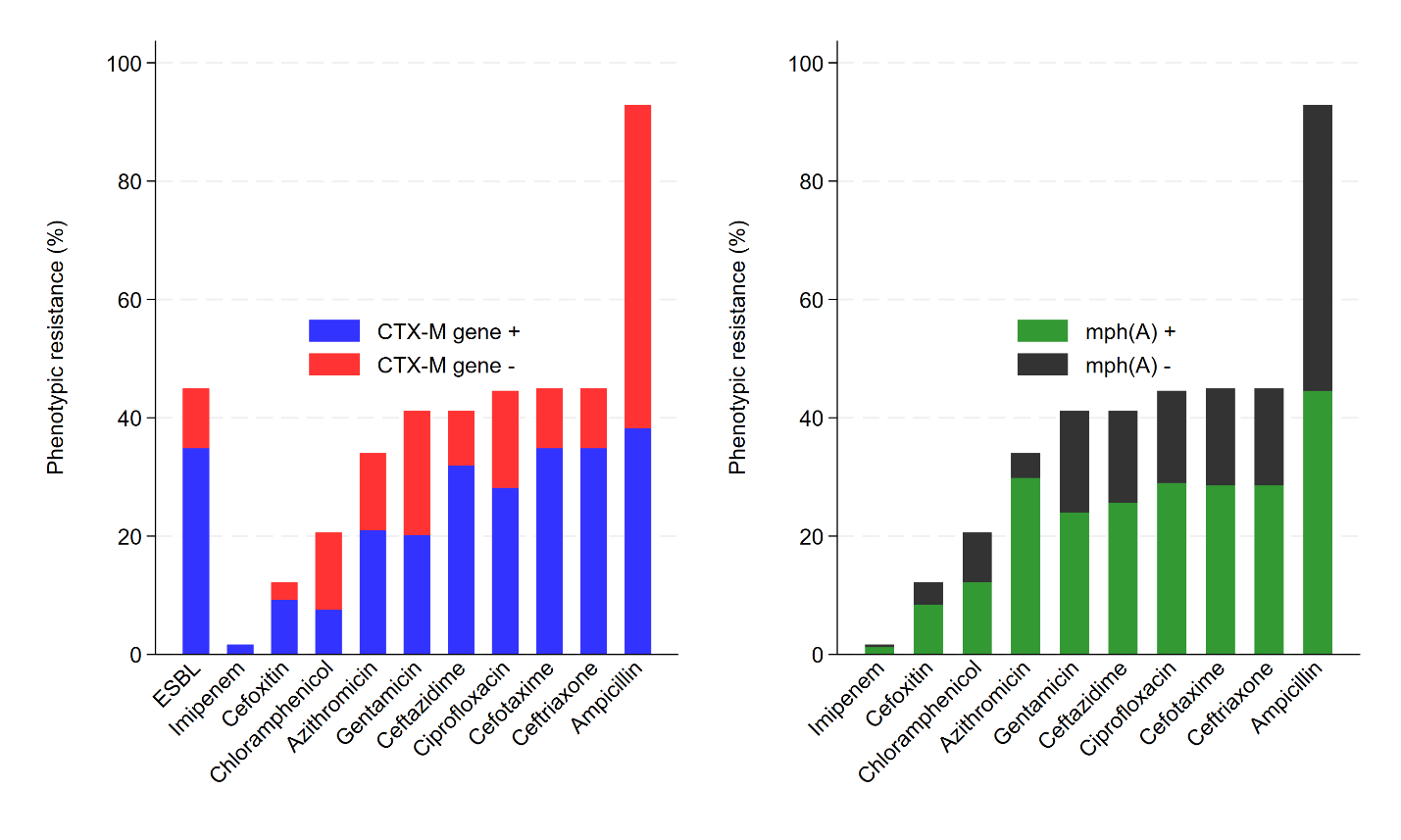


**A**

**B**

**Supplementary Figure 3**: Prevalence of phenotypic resistance in *E. coli* isolates from children discharged from hospital in western Kenya. Panels A and B show the prevalence of phenotypic resistance and the corresponding proportion of the children whose sample isolates had at least one genetic marker of β-lactamase and macrolide resistance respectively. Phenotypic resistance was determined using the disc diffusion technique and a child was deemed to have phenotypic resistance if any distinct morphology of *E. coli* from the child’s stool sample was resistant for a given antibiotic.


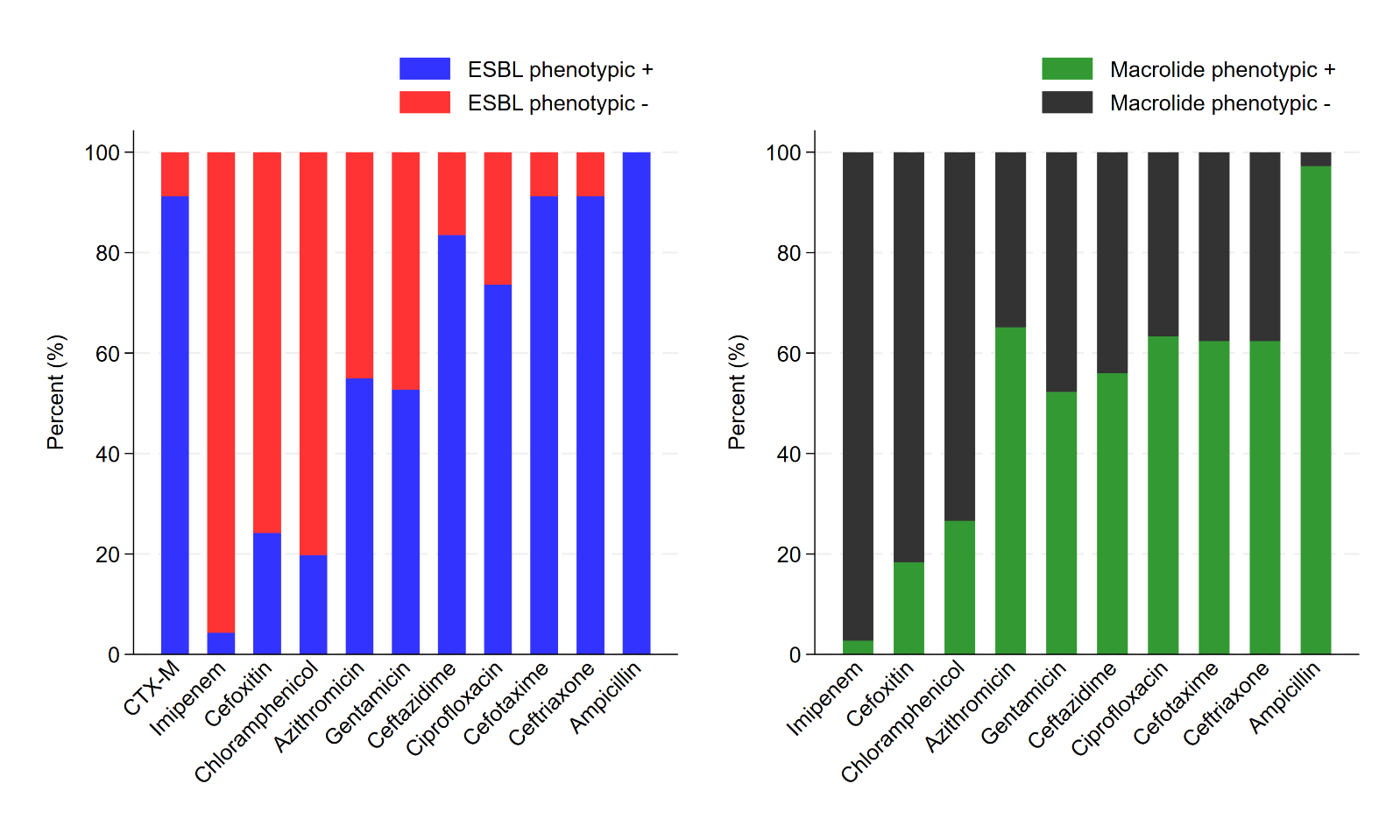


**B**

**A**

**Supplementary Figure 4**: Stack plots presenting the proportion of children whose *E. coli* isolates demonstrated phenotypic resistance among isolates that had bla_CTX-M_ and mph(A) genes. Panels A and B show the distribution of phenotypic resistance among sample isolates with bla_CTX-M_ and mph(A) respectively. Phenotypic resistance was determined using the disc diffusion technique. and a child was deemed to have phenotypic resistance if any distinct morphology of *E. coli* from the child’s stool sample was resistant for a given antibiotic.
